# Dyslipidemia treatment of patients with diabetes mellitus in a US managed care plan: a retrospective database analysis

**DOI:** 10.1186/1475-2840-8-26

**Published:** 2009-05-18

**Authors:** Peter P Toth, Victoria Zarotsky, Jane M Sullivan, Dave Laitinen

**Affiliations:** 1 Sterling Rock Falls Clinic, Sterling, IL, USA; 2 University of Illinois College of Medicine, Peoria, Illinois, USA; 3 Southern Illinois University School of Medicine, Springfield, Illinois, USA; 4 i3 Innovus, Eden Prairie, Minnesota, USA; 5 Abbott Laboratories, Abbott Park, Illinois, USA

## Abstract

**Background:**

To evaluate real-world pharmacologic treatment of mixed dyslipidemia in patients with diabetes mellitus (DM).

**Methods:**

All commercial health plan members in a large US managed care database with complete lipid panel results (HDL-C, LDL-C, TG) between 1/1/2006 and 12/31/2006 were identified (N = 529,236). DM patients (N = 53,679) with mixed dyslipidemia were defined as having any 2 suboptimal lipid parameters (N = 28,728). Lipid treatment status 6 months pre- and post-index date was determined using pharmacy claims for any lipid therapy.

**Results:**

Post-index, 41.1% of DM patients with 2 abnormal lipid parameters and 45.1% with 3 abnormal lipid parameters did not receive lipid-modifying treatment. Post-index treatment rates were 57.4%, 63.6%, and 66.4% for patients with LDL-C, HDL-C, and TG in the most severe quartiles, respectively. Statin monotherapy was the primary lipid-modifying regimen prescribed (54.8% and 47.8% of patients with any 2 and all 3 lipids not at goal, respectively). Less than 30% of treated patients received combination therapy.

**Conclusion:**

Over 40% of DM patients with mixed dyslipidemia received no lipid-modifying therapy during the follow-up period. Those who were treated were primarily prescribed statin monotherapy. This study suggests that DM patients are not being treated to ADA-suggested targets.

## Introduction

Cardiovascular disease (CVD) is a primary cause of morbidity and mortality among patients with hyperglycemia or type 2 diabetes mellitus (DM) despite the availability of effective therapies to treat major risk factors such as elevated blood pressure and cholesterol levels [1,2]. The most common lipid pattern in patients with DM, hypertension, and/or the metabolic syndrome includes hypertriglyceridemia, increased concentrations of small dense low-density lipoprotein particles, low levels of high-density lipoprotein cholesterol (HDL-C), increased remnant lipoproteins, and elevated apolipoprotein B concentrations [3,4]. This profile of mixed dyslipidemia significantly increases risk for all forms of atherosclerotic disease, including coronary heart disease (CHD) [5-8].

The burgeoning prevalence of insulin resistance throughout the world is greatly increasing the incidence of mixed dyslipidemia. It is estimated that approximately 40% of patients with coronary artery disease have low-density lipoprotein cholesterol (LDL-C) levels below 130 mg/dL yet these patients also have low levels of HDL-C, with or without increased levels of triglycerides (TG) [9,10]. Overall, isolated low HDL-C affects 20% to 30% of patients with CHD, representing several million people in the United States [9,10].

Observational studies indicate that low HDL-C levels are strongly and independently associated with increased CHD risk [11-13]. The results of the first major clinical trial specifically focusing on the treatment of low HDL-C demonstrated that lipid treatment that raised HDL-C and lowered TG but had no effect on LDL-C substantially reduced the incidence of major cardiac and cerebrovascular events [14].

Elevated serum levels of TG are an independent risk factor for CHD even after adjustment for HDL-C [7]. In a subanalysis of the Pravastatin or Atorvastatin Evaluation and Infection Therapy-Thrombolysis In Myocardial Infarction 22 (PROVE-IT TIMI 22) trial to assess the impact of low on-treatment TG on CHD risk beyond LDL-C < 70 mg/dL, Miller et al. [15] demonstrated that on-treatment TG below 150 mg/dL was independently associated with a lower risk of recurrent CHD events. The Veterans Affairs HDL Intervention Trial (VA-HIT), which included patients with low HDL-C (mean 31 mg/dL) and LDL-C (mean 111 mg/dL), found that patients receiving gemfibrozil had a 22% relative risk reduction in the primary end point of time to first nonfatal myocardial infarction or CHD death (95% CI 7% to 35%, p = 0.006). There was also a significant reduction in cerebrovascular events. Among 627 patients with DM, there was a 24% relative risk reduction for the expanded end point (CHD-related death, nonfatal myocardial infarction, or definite stroke). Likewise, among 1,449 patients with ≥ 3 criteria that define the metabolic syndrome (impaired fasting glucose; hypertension; obesity; high TG; or low HDL-C), the relative risk reduction with gemfibrozil was an even more impressive 35% for the expanded end point [14]. Together with data from the Helsinki Heart Study [16] and a subgroup analysis from the Bezafibrate Infarction Prevention study [17], these data suggest that fibrates may be particularly effective in treating dyslipidemia in patients with the metabolic syndrome.

Multiple guidelines writing groups advocate combination therapy for the management of multiple lipid abnormalities (NCEP ATPIII, ADA, AHA/ACC) [18,19]. Statin-fibrate combination therapy has been found to be more successful than statin monotherapy in achieving therapeutic targets in dyslipidemic patients with DM or the metabolic syndrome [20-22]. The ongoing Action to Control Cardiovascular Risk in Diabetes (ACCORD) trial was designed to evaluate the effect of simvastatin-fenofibrate combination therapy on cardiovascular risk in patients with type 2 DM [23]. The effect of statin-niacin combination therapy on atherosclerosis progression in patients with high cardiovascular risk (CHD and low HDL-C levels) has been evaluated in both the Arterial Biology for the Investigation of the Treatment Effects of Reducing Cholesterol 2 (ARBITER 2) and the HDL Atherosclerosis Treatment (HATS) studies. These trials demonstrated that the use of statin-niacin combination therapy is associated with stabilization of atherosclerotic disease.

Despite the abundance of data confirming the role of suboptimal lipoprotein levels as a risk factor for CHD and the availability of guidelines enumerating therapeutic lifestyle modifications and pharmaceutical treatment options for the management of mixed dyslipidemia, a substantial proportion of dyslipidemic patients remain untreated or inappropriately treated [24,25], or patients choose to discontinue treatment soon after it is initiated [26]. The primary objective of the present study was to achieve greater understanding of treatment patterns in "real-world" patients by evaluating pharmacologic treatment of DM patients with mixed dyslipidemia enrolled in a commercial health plan. Specifically, the purpose was to determine the proportion of DM patients with suboptimal LDL, HDL-C, and/or TG values not being appropriately treated with lipid-modifying medications.

## Methods

### Data Source

This was a retrospective claims data analysis using medical and pharmacy data, laboratory results, and enrollment information from a large managed health care plan in the United States, with the largest concentration of patients being in the southern and midwestern regions. Claims for services provided to members of this health plan are submitted for payment by physicians, facilities, and pharmacies. At the time the study was conducted, the administrative claims database included data for approximately 14 million health plan enrollees with both medical and pharmacy benefits. All study data were de-identified and accessed with protocols compliant with the Health Insurance Portability and Accountability Act. Institutional Review Board approval was therefore not required for this study.

### Study Subject Identification

This study was conducted to determine lipid treatment patterns in DM patients with mixed dyslipidemia. Study patients included commercial health plan enrollees with a laboratory value for LDL-C, HDL-C, and TG all drawn on the same day during the time period from January 1, 2006 through December 31, 2006. An index date was set as of the date of the first suboptimal test result or first optimal test result. Patients were required to have been continuously enrolled for 182 days prior to and 182 days following the index date.

Two groups of patients were created: a subset with all lipid values (LDL-C, HDL-C, and TG) under control (categorized as the "optimal cohort") and another subset with at least 1 suboptimal lipid value ("suboptimal cohort"). The criteria for the suboptimal cohort were developed in accordance with the National Cholesterol Education Program (NCEP) Adult Treatment Panel (ATP) III [19] and American Heart Association (AHA) guidelines [13-15]. For DM/CHD patients, suboptimal lipid values were defined as LDL-C ≥ 100 mg/dL, HDL-C ≤ 40 mg/dL for males and ≤ 50 mg/dL for females, and TG ≥ 150 mg/dL.

### Study Measures

Patient demographic variables (age, gender, and geographic location) were captured from the enrollment data. Lipid lab values (LDL-C, HDL-C, TG) were obtained from the lab results data. Study patients were observed for 182 days prior to the index date (pre-index period) and for 182 days after the index date (post-index period) to determine their lipid risk factors for CVD and their pharmaceutical prescription patterns. For the assignment of LDL-C goal, patients were classified into 4 risk categories: (1) age/gender (male, ≥ 45 years; female, ≥ 55 years); (2) CHD (a medical claim indicating presence of CHD during the pre-index period); (3) hypertension (presence of ICD-9-CM code 401.x–404.x, 642.0x–642.2x, 642.7x during the pre-index period); and (4) DM (presence of ICD-9-CM code 250.xx or at least 2 filled prescriptions for oral antidiabetic agents during the pre-index period).

The presence of a pharmacy claim for any lipid therapy, including statins, fibrates, niacin, or various combinations, was determined. Specifically, separate determinations were made to identify patients with the following prescription patterns during the pre-index and post-index periods: (1) pharmacy claim for a statin (atorvastatin, cerivastatin, fluvastatin, lovastatin, pravastatin, rosuvastatin, simvastatin, or atorvastatin + amlodipine [Caduet^®^]) and without a claim for other lipid-modifying medications; (2) pharmacy claim for a fibrate (clofibrate, gemfibrozil, fenofibrate) and without a claim for other lipid-modifying medications; (3) pharmacy claim for a niacin/nicotinic acid and without a claim for other lipid-modifying medications; (4) pharmacy claim for "other" medication (cholestyramine, colestipol, colesevelam, ezetimibe) and without a claim for other lipid-modifying medications; (5) pharmacy claim for a statin and a fibrate; (6) pharmacy claim for a statin and niacin/nicotinic acid; (7) pharmacy claim for a statin and other lipid-modifying medication; (8) pharmacy claim for a fibrate and a niacin; (9) pharmacy claim for a fibrate and other medications (cholestyramine, colestipol, colesevelam, ezetimibe); (10) pharmacy claim for a niacin and other medications (cholestyramine, colestipol, colesevelam, ezetimibe); (11) combinations other than those already mentioned; and (12) no claim for a lipid-modifying medication.

### Analysis

All study variables were analyzed descriptively. Numbers and percentages are provided for dichotomous and polychotomous variables. Means, medians, standard deviations, and percentiles are provided for continuous variables. Differences in mean values were assessed using a t-test and differences in proportions were assessed using a chi-squared test.

## Results

### Patient Characteristics

Of the 3.9 million health plan enrollees in 2006, lab values for LDL-C, HDL-C, and TG levels were available for a total of 529,236 patients. Of these, 65,242 (12.3%) met the definition of having DM, and 82.3% (n = 53,679) of the DM patients had at least 1 suboptimal lipid value and 17.7% (n = 11,563) had optimal values for all lipid tests The mean age of the study population was 52 years, and was similar in the suboptimal cohort and optimal cohort (p = 0.0725). Fifty-two percent of patients were female and 47% were male in the study population (Table 1).

**Table 1:** Demographic Information for Patients with Diabetes

Demographics	Total (N = 65,242)		Suboptimal Cohort (N = 53,679)		Optimal Cohort (N = 11,563)		p-value
	mean	std	mean	std	mean	std	

Age (continuous)	52.43	10.53	52.39	10.22	52.61	11.86	0.0725

	n	%	n	%	n	%	

Age							< 0.0001

< 45(M) or < 55(F)	22,697	34.79	19,011	35.42	3.686	31.88	

> = 45(M) or > = 55(F)	42,545	65.21	34,668	64.58	7,877	68.12	

Gender							< 0.0001

Male	34,538	52.94	27,695	51.59	6,843	59.18	

Female	30,704	47.06	25,984	48.41	4,720	40.82	

Geographic Location							< 0.0001

Northeast	8,695	13.33	7,061	13.15	1,634	14.13	

Midwest	13,374	20.50	10,979	20.45	2,395	20.71	

South	40,440	61.98	33,489	62.39	6,951	60.11	

West	2,733	4.19	2,150	4.01	583	5.04	

% is calculated from the Column total

Among the diabetes patients in the suboptimal cohort (n = 53,679), approximately 54% (n = 28,728) had more than 1 lipid abnormality. The lipid abnormalities in the DM population were as follows: 23% had high LDL-C values only, 13% had low HDL-C only, 10% had elevated TG only, 10% had high LDL-C and low HDL-C, 12% had elevated LDL-C and TG, 16% had low HDL-C and elevated TG, and 16% had abnormal values for all 3 lipid fractions.

### Treatments

**Figure 1:**
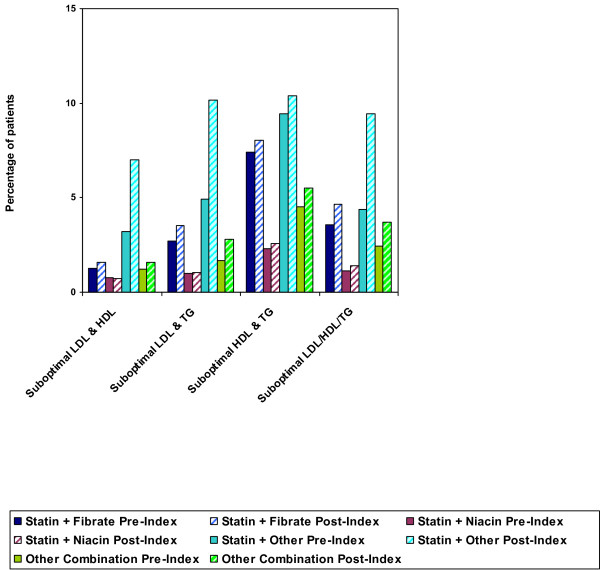
**DM Patients (n = 28,728) with > 1 Suboptimal Lipid Value Not Taking Any Lipid-Modifying Therapy, Pre- vs. Post-Index**.

Overall, an increase in lipid-modifying therapy from the pre-index period to the post-index period was observed in DM patients with mixed dyslipidemia. In the pre-index period, 68% of DM patients with suboptimal LDL-C and HDL-C, 58% with suboptimal LDL-C and TG, 32% with suboptimal HDL-C and TG, and 60% with suboptimal values for all 3 lipid parameters received no treatment for dyslipidemia. In the post-index period, the percentages of patients not receiving therapy decreased to 58%, 43%, 29%, and 45% in each group, respectively. Patients with suboptimal HDL-C and TG had the smallest decrease in the percentage of patients not receiving lipid-modifying therapy (Figure 1).

**Figure 2:**
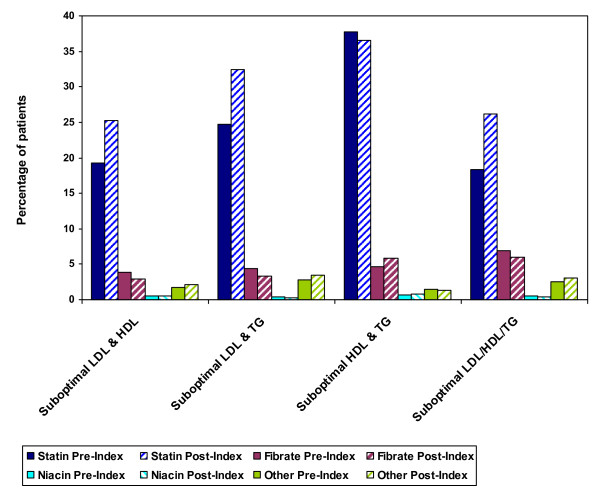
**Monotherapy Patterns for DM Patients with > 1 Suboptimal Lipid Value, Pre-Index vs. Post-Index**.

**Figure 3:**
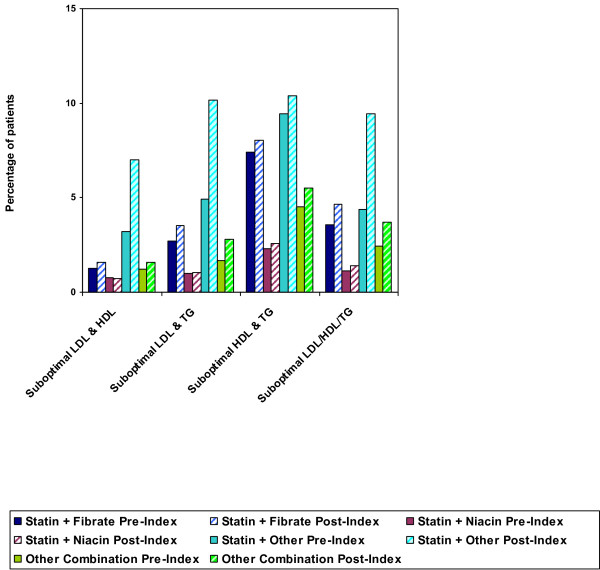
**Combination Therapy Patterns for DM Patients with > 1 Suboptimal Lipid Value, Pre-Index vs. Post-Index**.

Among DM patients with more than 1 lipid abnormality, 25% to 37% were prescribed statin monotherapy, 3–6% received fibrate monotherapy, and less than 1% received niacin monotherapy during the post-index period. Figure 2 illustrates the pre-index versus post-index monotherapy patterns for DM patients with mixed dyslipidemia. The proportion of DM patients treated with statins in combination with other lipid-modifying drugs increased from the pre-index to post-index time period but still remained low (1.5% to 3.5% of patients receiving statin and fibrate therapy, < 1.0% to 2.6% of those receiving statin and niacin therapy, and 7.0% to 10.4% of those receiving statin and other lipid-modifying therapy). Figure 3 shows the pre-index versus post-index combination treatment patterns for this patient group.

**Figure 4:**
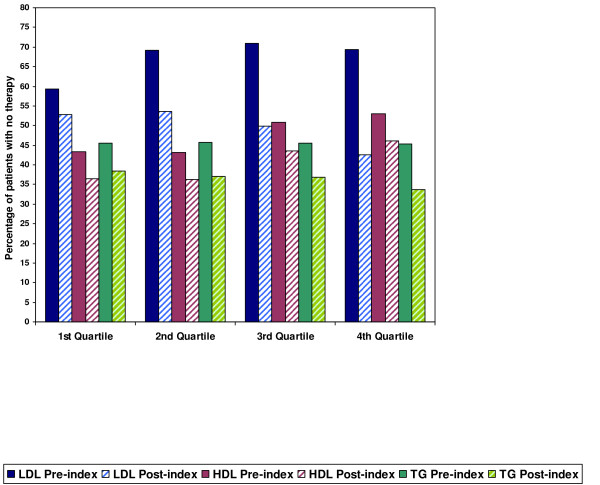
**DM Patients Not Receiving Lipid-Modifying Treatment, by Quartiles of LDL-C, HDL-C, and TG Value, Pre-Index vs. Post-Index**.

The treatment patterns for patients with DM were also evaluated by quartiles of suboptimal LDL-C, HDL-C, and TG values. The ranges for the quartiles are defined in Figure 4. A total of 32,855 patients (61%) were identified as having elevated LDL-C, 29,398 (55%) had low HDL-C, and 28,755 (54%) had elevated TG. Among the patients with a suboptimal lipid value and DM, the percentages of patients not receiving treatment were 63%, 47%, and 49%, respectively. The percentage of patients not receiving lipid-modifying therapy decreased in the post-index period, but remained high in every quartile as illustrated in Figure 4. In the first quartile, the percentages of patients not receiving treatment for suboptimal LDL-C, HDL-C, and TG were 53%, 36%, and 39%, respectively. In the fourth quartile, the percentage of patients not receiving therapy decreased to 43% for patients with suboptimal LDL levels, but increased for patients with suboptimal HDL-C and TG (to 46% and 40%, respectively). When treatment was initiated, statin monotherapy was the most commonly prescribed regardless of the type of lipid abnormality and the quartile.

## Discussion

Despite the availability of treatment guidelines for dyslipidemia management and the abundance of clinical trial evidence highlighting the benefits of lipid-modifying therapy to reduce the risk for CHD and its clinical sequelae, treatment of DM patients with abnormal LDL-C, HDL-C, and TG levels continues to be suboptimal. In our patient population, 16% of patients with suboptimal lipid values and 21% of our mixed dyslipidemia group had DM. Our DM patient population had a high (> 50%) proportion of patients with abnormal HDL-C and TG levels. This is in line with the results of a recent study by Grant et al. [27], who also found a high prevalence of suboptimal HDL-C among patients with type 2 diabetes, with nearly half (49.5%) of patients exhibiting low HDL-C. Another study, involving a high-risk population with documented CHD or CHD risk equivalents, also found a high prevalence of low HDL-C across various LDL-C levels, including among patients taking statins [28]. In that study, low HDL-C was most prevalent in patients with LDL-C of 70 mg/dL or lower, and was equally and highly prevalent in patients taking statins (67%) and those not taking statins (64%).

A large percentage of DM patients with suboptimal lipid levels in our study did not receive lipid-modifying therapy either prior to or after a suboptimal lipid value was obtained (53% and 42%, respectively). While the percentage of patients receiving lipid-modifying therapy increased in the post-index period, a substantial number of DM patients with suboptimal lipid levels did not receive any lipid-modifying therapy in the post-index period irrespective of abnormal lipid parameter, or the degree of the abnormal value. At least 30% of DM patients with suboptimal lipid levels across all mixed dyslipidemic groups were not treated with any lipid-modifying medication for at least 6 months post-index. Although lipid treatment rates increased post-index in all mixed dyslipidemic groups, and even though treatment rates were correlated with the number of total risk factors, abnormal lipid parameters and more severe lipid value quartiles, the incremental use of lipid-modifying medication was not as substantial as would be expected, given the compelling association between multiple lipid abnormalities and cardiovascular risk in patients with DM. Of particular note is that in DM patients with low HDL-C and elevated TG, the most common post-index lipid therapy used was statin monotherapy (in 37% of patients), and only 17% received any niacin or fibrate therapy to target suboptimal HDL-C or TG. Grant et al. [27] described similar findings, with the vast majority of their study patients receiving statin monotherapy and a very small percentage being prescribed treatment targeting the suboptimal HDL-C level.

These results are also consistent with those reported by Klingman and colleagues [29], who evaluated data from the 1999–2000 National Health and Nutrition Examination Survey to assess the state of dyslipidemia management in the US adult population and to determine whether treatment patterns were consistent with guideline recommendations. They demonstrated that 44% of treatment-eligible adults had never been told by a physician, or any other healthcare professional, that they had dyslipidemia. Among all treatment-eligible adults, only 30% were adopting therapeutic lifestyle changes (TLC) and only 10% were receiving lipid-modifying therapy. Very high percentages of treatment-eligible adults were receiving no treatment at all for dyslipidemia: 69% overall, 61% of high-risk adults, 70% of medium-risk adults, and 77% of low-risk adults. Unlike the present study, however, Klingman and colleagues focused on the achievement of LDL-C goals and did not evaluate HDL-C and TG levels.

Although the reasons for the suboptimal treatment of dyslipidemia are beyond the scope of this study, several explanations have been proposed to account for the discrepancy between guideline recommendations and real-world treatment patterns. These include suboptimal patient-follow-up, use of low-potency statins in high-risk patients, inaccurate pill counts and refill records, patients' inability to pay for medications, lack of patient motivation [30], physician time constraints, difficulty understanding and applying NCEP guidelines [19], and lingering concerns about the potential hepatic and skeletal muscle toxicity of combinations of lipid-modifying drugs [31].

NCEP ATPIII recommends adjunctive therapy with niacin or fibrates (to achieve non-HDL goal) for dyslipidemic patients with multiple CVD risk factors who have low HDL-C and/or high TG, after the LDL-C goal has been achieved with statin monotherapy [7,19,32]. Our study results suggest that the majority of patients with DM receiving lipid-modifying treatment are being treated with statin monotherapy. Although it is well documented that the different classes of lipid-modifying medications have different (complementary) effects on lipid parameters [19], statin monotherapy continued to be prescribed to 81% of patients with more than 1 suboptimal lipid value who received treatment during the post-index period of our study, even though many patients with multiple lipid abnormalities – i.e., those with suboptimal HDL-C and TG levels – may have gained greater benefit from adjunctive treatment with either niacin, a drug with the greatest capacity to increase HDL-C, or fibrates, which have the most pronounced capacity to decrease elevations in serum TG. This is despite the fact that NCEP ATPIII was published in 2001, 5 years prior to the conduct of this study [19].

Our findings regarding prescribing habits for mixed dyslipidemia are consistent with results reported by Stacy and colleagues [33], who evaluated 600 high-volume prescribers of lipid-modifying drugs in 6 metropolitan areas identified from the IMS Health prescription database. Their study also found that 40% of the study population potentially had mixed dyslipidemia. These investigators reported that a high percentage of patients (69%) were prescribed monotherapy with lipid-modifying medication, of which 82% was statin monotherapy; furthermore, 14% were not prescribed any lipid-modifying drug therapy.

### Study Limitations

The findings of this study must be considered within the limitations of the data and study design. Claims data are collected for the purpose of payment and not research and may not accurately capture a patient's medical use history. While these data provide insight into real-world treatment patterns, they are subject to possible coding errors. Certain information is not readily available in claims data that could have an effect on study outcomes, such as certain clinical and disease-specific parameters.

Furthermore, the presence of a prescription claim does not necessarily indicate that the drug was taken or that it was taken as prescribed – nor does it reflect those patients who may have received drugs without the presence of a prescription claim either by receiving drug samples or filling a prescription outside of the health care pharmacy system. The presence of a diagnosis code on a medical claim does not demonstrate positive presence of disease, as it may be incorrectly coded or included as a rule-out criterion rather than as an indication of actual disease.

In addition, because patients were identified from a managed care plan, this study may not be generalizable to the general population and may not be applicable to a setting outside of managed care. Finally, a limitation specific to this study is that the description for values in the laboratory results does not clearly indicate whether lipid values were obtained during fasting. Although these limitations do not reduce the strength of the study, they must be considered when interpreting the results.

## Conclusion

In real-world clinical practice, pharmacologic treatment rates increased only slightly upon assessment of multiple lipid abnormalities and in patients with DM. Over 42% of DM patients with mixed dyslipidemia received no lipid-modifying therapy after suboptimal lipid levels were ascertained, and those patients who were treated, even those with low HDL-C and high TG levels who may benefit from adjunctive treatment with niacin or fibrates, were primarily prescribed statin monotherapy. These study results suggest DM patients are not being treated to ADA-suggested targets. Further research is warranted to investigate how physicians tailor lipid-modifying therapy in these patients so that solutions aimed at improving treatment rates can be devised in order to help DM patients better achieve guideline-defined LDL-C, HDL-C, and TG goals, thereby potentially reducing their risk for cardiovascular events.

## Competing interests

DL works for Abbott Laboratories, which provided the financial support to sponsor the research for this study. PT received an honorarium from Abbott Laboratories for consulting services on this study and development of this manuscript.

## Authors' contributions

DL conceived of the study and participated in its design and coordination. PT participated in the design of the study and helped draft the manuscript. JS participated in the design of the study and performed the statistical analysis and programming. VZ participated in the design and coordination of the study and drafted the manuscript. All authors read and approved the final manuscript.
